# Integrated Physioecological Adaptation of *Apodemus chevrieri* to Water Deprivation: Synergistic Responses in Body Mass Regulation and Water Metabolism

**DOI:** 10.1093/iob/obag055

**Published:** 2026-09-21

**Authors:** D Liu, W Zhu

**Affiliations:** School of Life Sciences, Yunnan Normal University, Kunming, Yunnan 650500, China; School of Life Sciences, Yunnan Normal University, Kunming, Yunnan 650500, China

## Abstract

As global climate change intensifies, increasing drought in Southwest China may impose strong water stress on small mammals in the Hengduan Mountains. *Apodemus chevrieri* is endemic to this region, but its integrated responses to water restriction remain unclear. Here, we compared control and water-stressed individuals and quantified changes in body mass, food intake, body temperature (*T*_b_), metabolic traits, evaporative water loss (EWL), metabolic water production relative to EWL (MWP/EWL), and visceral and digestive-tract morphology. Water restriction reduced body mass, food intake, core *T*_b_, and metabolic rate. It also reduced small intestine mass, but increased the size of the large intestine and cecum, indicating enhanced hindgut development. EWL increased with rising ambient temperature (*T*_a_), while MWP/EWL decreased as *T*_a_ increased. Water deprivation shifted the *T*_a_ at the water balance point (*T*_a_ @ MWP = EWL) from 12.5 to 16°C. These results indicate that under water deprivation stress, *A. chevrieri* reduces water and energy expenditure by lowering body mass and *T*_b_, downregulating metabolic rate, and enhancing hindgut structure to improve water reabsorption. Interspecific comparisons further suggest that although this species generally exhibits water physiological characteristics typical of mesic habitats, it shows physiological plasticity consistent with a partial shift toward a xeric water-economy strategy under water deprivation stress. This integrated strategy reveals the adaptive potential of *A. chevrieri* to seasonal drought in the Hengduan Mountains.

## Abstract (zh)

随着全球气候变化加剧, 中国西南地区日益严重的干旱可能对横断山区小型哺乳动物造成强烈的水分胁迫。高山姬鼠(*Apodemus chevrieri*) 是该区域的特有种, 但其对水分限制的整合性响应尚不清楚。本研究比较了对照组与缺水胁迫组个体, 测定并分析了体重、摄食量、体温(T_b_) 、代谢特征、蒸发失水(EWL) 、代谢产水与蒸发失水的比值(MWP/EWL) 以及内脏和消化道形态的变化。结果表明, 水分限制导致体重、摄食量、核心体温和代谢率下降;同时小肠质量减少, 但大肠和盲肠体积增大, 表明后肠发育增强。蒸发失水随环境温度(T_a_) 升高而增加, 而MWP/EWL 随T_a_ 升高而降低。水分剥夺使水分平衡点对应的环境温度(T_a_ @ MWP = EWL) 从12.5°C 偏移至16°C 。这些结果表明, 在缺水胁迫下, 高山姬鼠通过降低体重和体温、下调代谢率以及增强后肠结构以提高水分重吸收, 从而减少水分和能量消耗。种间比较进一步表明, 尽管该物种总体上表现出典型湿润生境的水分生理特征, 但在缺水胁迫下, 其表现出向干旱生境水经济策略部分转变的生理可塑性。这一整合策略揭示了高山姬鼠对横断山区季节性干旱的适应潜力。

## Introduction

Global climate warming is driving shifts in precipitation patterns by accelerating glacial melt and intensifying the atmospheric water cycle, thereby profoundly impacting terrestrial ecosystems (Schewe et al. 2014; IPCC 2021). While some regions are becoming wetter (Nicholson et al. 2018; Lu et al. 2021), southwestern China has shown a clear increase in drought because of reduced precipitation and rising temperature (Wang et al. 2020). These regional hydrological shifts, characterized by declining annual precipitation and more frequent seasonal water shortages, create new physiological challenges for the survival and adaptation of small mammals. Water is fundamental to life and is a major environmental factor shaping broad-scale patterns of species distribution and biodiversity (Hawkins et al. 2003; Chaplin 2006). As a core factor for survival, water availability directly regulates physiological functions, energy allocation, and ecological adaptation strategies (Hinds and MacMillen 1985). For small mammals, coping with limited water availability often requires integrated physiological adjustments. Rather than relying on a single mechanism, small mammals inhabiting arid or increasingly dry environments exhibit coordinated physiological flexibilities, such as optimizing water intake through dietary shifts, enhancing metabolic water production, and improving renal osmotic regulation (Blumstein et al. 2025; Yuan et al. 2025). Furthermore, *Orientallactaga sibirica* alters its gut microbiota in ways that may contribute to adaptation to water-limited environments (Zhang et al. 2025). These responses suggest that water limitation can influence energy allocation and hydration balance in small mammals.

The physiological ecology of water balance has been studied extensively in xeric-adapted rodents such as *Dipodomys spp.* and *Peromyscus eremicus*, revealing specialized mechanisms, including highly efficient renal concentration, reduced evaporative water loss (EWL), and elevated metabolic water production, that allow survival in arid environments (McNab 1979; MacMillen and Hinds 1983; Hinds and MacMillen 1985; Blumstein and MacManes 2023). However, comparatively less is known about whether species from mesic habitats possess the physiological plasticity to shift their water-economy phenotypes toward xeric-like strategies when confronted with acute water deprivation.

For small mammals, thermoregulation, body-mass regulation, and water balance are functionally linked under water-limiting conditions. Water contributes to heat exchange through evaporation and is also essential for digestion, nutrient transport and absorption, and waste excretion (Robertshaw 2006; Jéquier and Constant 2010). Under resource-limited conditions, small mammals can cope with external stressors by reducing body mass, suppressing energy expenditure, and modulating endocrine levels (Zhu et al. 2014). A common pattern across studies is that water deprivation specifically modifies maintenance metabolism and heat dissipation in directions that conserve both water and energy (Wunder 1970; Blumstein and MacManes 2023; Yang and Zhu 2025). Therefore, water shortage plays a key role in body mass regulation by disrupting water and salt balance, suppressing food intake, and altering digestive metabolism, either directly through fluctuations in body water content or indirectly through reallocation of the energy budget.

Digestive-tract flexibility is another important mechanism by which small mammals respond to water shortage (Wang et al. 1995). Unlike gut microbiota modulation, which operates through microbial community shifts (Zhang et al. 2025), digestive organ phenotypes reflect direct structural adjustments in tissue mass and surface area that alter nutrient and water processing capacities (Pucek 1965; Zhu et al. 2012). The gastrointestinal tract regulates the absorption and metabolism of nutrients through complex mechanical, chemical, and neuroendocrine mechanisms, with the large intestine being the main site of water and electrolyte reabsorption (Kunzelmann and Mall 2002). Because most studies of small-mammal water balance have emphasized pulmonary and cutaneous EWL (Xi and Sun 1973; Wang et al. 1993; Zhu et al. 2008), less is known about how digestive morphology is coordinated with metabolic and thermoregulatory responses under acute water deprivation.

*Apodemus chevrieri* (Rodentia, Murinae, and Apodemus) is a typical Palearctic rodent endemic to the mountain forests of the Yunnan–Guizhou Plateau (Zhu et al. 2016). The Hengduan Mountain area in western Yunnan, where this species occurs, is characterized by complex topography, stable annual temperature regimes, and remarkable biodiversity (Zhang et al. 2017; Chen et al. 2022). As this region experiences an escalating trend of seasonal droughts with reduced precipitation and higher temperatures, understanding how this mesic-adapted endemic species acclimates to sudden water shortages is crucial for predicting population dynamics. Our laboratory has extensively characterized its baseline physiological responses, including thermogenesis, photoperiodic adaptations, and EWL circadian rhythms under ad libitum conditions (Luo et al. 2011; Zhu and Wang 2012; Zhu et al. 2013b; Gao and Zhu 2024). However, it remains unclear how this mesic-adapted endemic species coordinates its energy metabolism, body mass, and digestive tract morphology under acute water deprivation, and whether it can flexibly shift its water-economy phenotype toward that of xeric-adapted rodents.

To address this, we tested two main hypotheses regarding the physiological plasticity of *A. chevrieri* under short-term water deprivation. First, we hypothesized that water-deprived individuals will adopt an integrated water-saving strategy by reducing energy expenditure and maximizing water retention. Specifically, we predicted that water restriction would lead to reduced food intake, body mass, *T*_b_, and metabolic rate, alongside morphological enhancement of hindgut traits associated with water reabsorption. Second, we hypothesized that acute water deprivation would induce phenotypic flexibility allowing this mesic species to functionally approach the water-economy traits of xeric species. By placing *A. chevrieri* in a cross-habitat rodent comparison, we predicted that hydrated individuals would exhibit a metabolic water production to evaporative water loss ratio (MWP/EWL) typical of mesic rodents, whereas water deprivation would shift its MWP/EWL threshold toward values observed in xeric rodents without necessarily reaching the level of specialized desert species. These findings will deepen our understanding of the survival and adaptive strategies of *A. chevrieri* and provide a robust scientific basis for predicting the responses of small mammal population dynamics to climate change in the Hengduan Mountains.

## Materials and methods

### Experimental animals

*Apodemus chevrieri* were captured in Chenggong District, Kunming City, Yunnan Province, in July 2025 (24° 52′ 4″ N, 102° 51′ 57″ E, altitude 2020 m), a low-latitude plateau mountain monsoon region in the northern subtropical zone with an average annual temperature of about 16.5°C and an average maximum temperature of 25°C in July. Following disinfection and flea removal, the animals were transported to the animal facility at Yunnan Normal University. Individuals were housed individually in plastic cages (260 mm × 160 mm × 150 mm) without nesting material. The housing temperature was 25 ± 1°C, and the ambient light was 12L:12D. The animals used in this experiment were all adults in the non-reproductive period. Twelve healthy adult individuals with similar body weights were selected.

Two weeks after acclimation to laboratory conditions, *A. chevrieri* were randomly divided into two groups: the animals in the water-stressed group were provided with standard feed and denied access to water (*n* = 6, ♀:♂ = 4:2), and the control group was provided with standard feed and ad libitum water (*n* = 6, ♀:♂ = 5:1); room temperature was maintained at 25 ± 1°C, and the photoperiod was 12L:12D. The experiment lasted for 7 days. Body mass, *T*_b_, metabolic rate, and food intake of *A. chevrieri* were measured on days 0, 3, and 7, and an infrared thermal imager (WIC640-SUW; Workswell, Roznov, Czech Republic) was used for thermal imaging of *A. chevrieri*. At the end of the 7-day period, animals were euthanized using carbon dioxide gas, and tissues were collected for body composition and digestive tract analysis. There was no significant difference in body mass between the two groups before the experiment (*P* > 0.05). All animal procedures were in accordance with the regulations of the Medical Bioethics Committee of Yunnan Normal University and ethical standards (No. 13-0901-011).

### Measurement of body mass and food intake

Body mass of *A. chevrieri* was measured using an electronic balance (AB204-S, Switzerland, accuracy of 0.01 g) at 08:30 on the measurement day. Food intake of *A. chevrieri* was measured using the food balance method. Feeding was completed from 09:00 to 10:00 on the day before the measurement, and the amount of food provided was recorded. The remaining food was collected at the same time of day and dried in an oven, and the food residue was recorded. Intake = feeding amount − remaining amount (Zhu et al. 2011).

### Measurement of metabolic rate

Basal metabolic rate (BMR) of *A. chevrieri* was measured using an FMS portable respiratory metabolic measurement system (Sable Systems International, Inc.). Before the determination, the animals were fasted for 2–4 h. The animals were placed in the respiratory chamber with a volume of 1.5 L; the air pump flow rate was 200 mL/min, and *T*_a_ was controlled at 25.0 ± 0.5°C (within the thermal neutral zone) using a climate-controlled chamber (SPX-300, Shanghai Boxun Medical Equipment Factory) (Zhu et al. 2008). The animals were allowed to acclimate to the chamber for 30 min while resting. ExpeData software was then used to measure metabolic rate over four rounds, with each round lasting 15 min. Data were recorded once every 5 min for a total of 60 min. After the experiment, the data were exported, and the lowest 10 consecutive oxygen consumption values were selected as the BMR. The mean metabolic rate calculated over the entire 60-min measurement period was defined as overall metabolic rate.

### T_b_ and infrared thermography

Core *T*_b_ was measured using a digital thermometer (XGN-1000T, Beijing Yezhiheng Technology Co., Ltd.; accuracy, 0.1°C). Before measurement, the probe was disinfected and lubricated with petroleum jelly. The probe was then inserted approximately 2 cm into the rectum, and the reading was taken after 1 min. Each animal was measured three times, and the mean value was used for analysis.

Additionally, infrared thermographic images of the whole-body surface temperature distribution were captured using a thermal imager (WIC640-SUW; Workswell, Czech Republic) at a fixed distance of 1 m perpendicular to each animal while it was in a resting state. To ensure longitudinal comparability of the data, each individual was repeatedly imaged at each measurement time point (Days 0, 3, and 7), with all imaging sessions consistently conducted between 09:00 and 10:00 to minimize circadian effects on surface temperature. Maximum surface temperatures were subsequently extracted from the cranial and tail regions using the accompanying thermal analysis software (Tattersall and Cadena 2010).

### Determination of EWL and MWP/EWL ratio

To better evaluate the water-conservation capacity of *A. chevrieri* under water deprivation stress, EWL was measured at different *T*_a_ points after the metabolic rate test on day 7. A U-shaped tube containing color-changing silica gel was connected to the inlet of the respiratory chamber, and dry air was supplied to the chamber. A pre-weighed U-shaped tube containing dry silica gel was connected to the outlet (accuracy: 0.1 mg). Air was sampled for 15 min using a TMP1500 atmospheric sampling machine (Jiangsu Jintan Yitong Electronics Co., Ltd.), with the flow rate controlled at 0.5–1.0 L/min. Paraffin oil was placed at the bottom of the respiratory chamber to prevent the animals from urinating during the experiment. The mass difference of the desiccant before and after the experiment was used to calculate EWL (Zhu et al. 2008). The MWP/EWL ratio for each individual was calculated and used as the core index to evaluate water economy and water-regulation efficiency. MWP was converted using 1 mL O₂ = 0.62 mg metabolic water (Cortés et al. 2000).

### Measurement of body composition and digestive organs

After *A. chevrieri* were euthanized, the brain, heart, lungs, liver, and kidneys were carefully dissected. The whole brain was removed from the cranial cavity after opening the skull, and adhering meninges and surface moisture were gently removed before weighing. Adherent connective tissue and fat were removed, and blood on the organ surface was blotted dry with filter paper. The organs were then weighed to the nearest 0.001 g as wet organ masses. The digestive tract was removed, and the stomach, small intestine, large intestine, and cecum were separated. After other attached tissues were removed, surface moisture was blotted dry with filter paper, and each part was weighed. Length was then measured with a ruler to the nearest 0.01 cm.

### Data sources for comparative analysis

The EWL and *T*_a_ @ MWP = EWL data for rodents in different habitats used in the study are detailed in Table 1, including 25 species from mesic and xeric habitats. The physiological values compiled in Table 1 represent the mean values reported for each species under their respective hydrated experimental conditions.

**Table 1:** EWL and *T*_a_ @MWP = EWL values for rodent species from different habitats

Species	Habitat	Body mass (g)	EWL (mg/g h)	*T*_a_ @ MWP = EWL (°C)	Biogeographic region	Reference
*Abrothrix andinus*	Mesic	26.6	2.35	12.5	Talabre, Chile	Cortés et al. (2000)
*Abrothrix longipilis*	Mesic	52.6	2.4	9.5	Fray Jorge National Park, Chile	Cortés et al. (2000)
*Abrothrix olivaceus*	Mesic	21.6	2.21	14.1	Ojo Opache, Chile	Cortés et al. (2000)
*Eothenomys miletus*	Mesic	44.93	3.65	13.9	Hengduan Mountains, China	Zhu et al. (2008)
*Eothenomys olitor*	Mesic	34.26	3.17	12.96	Hengduan Mountains, China	Zhu et al. (2013a)
*Heteromys desmarestianus*	Mesic	75.8	1.51	18	Finca La Pacifica, Costa Rica	Cortés et al. (2000)
*Liomys irroratus*	Mesic	44.9	1.97	16.1	Edinburg, TX, USA	Cortés et al. (2000)
*Liomys salvani*	Mesic	42.7	2.38	14.4	Finca La Pacifica, Costa Rica	Cortés et al. (2000)
*Octodon lunatus*	Mesic	165.2	1.1	12.6	Las Chinchillas National Reserve, Chile	Cortés et al. (2000)
*Oligoryzomys longicaudatus*	Mesic	27.5	2.77	12.1	Fray Jorge National Park, Chile	Cortés et al. (2000)
*Phyllotis darwini*	Mesic	78	1.93	14	Fray Jorge National Park, Chile	Cortés et al. (2000)
*Phyllotis magister*	Mesic	53.5	2.08	10.5	Ojo Opache, Chile	Cortés et al. (2000)
*Phyllotis rupestris*	Mesic	63.6	2.15	12.1	Quebrada Jerez, Chile	Cortés et al. (2000)
*Chaetodipus baileyi*	Xeric	29.2	1.42	20.8	Pima County, AZ, USA	Hinds and MacMillen (1985)
*Chaetodipus flavus*	Xeric	8.4	1.9	18.9	Elgin, AZ, USA	Hinds and MacMillen (1985)
*Chaetodipus hispidus*	Xeric	32.1	1.93	16	Elgin, AZ, USA	Hinds and MacMillen (1985)
*Chinchilla lanigera*	Xeric	365	0.58	12.7	Las Chinchillas National Reserve, Chile	Cortés et al. (2000)
*Dipodomys deserti*	Xeric	104.7	1	16.2	Dale Dry Lake, CA, USA	Hinds and MacMillen (1985)
*Dipodomys merriami*	Xeric	35.8	1.7	17	Morongo Valley, CA, USA	Hinds and MacMillen (1985)
*Dipodomys ordii*	Xeric	46.8	1.33	18.7	Benton Valley, CA, USA	Hinds and MacMillen (1985)
*Dipodomys panamintinus*	Xeric	64.2	0.94	20.7	Mono County, California, USA	Hinds and MacMillen (1985)
*Microdipodops megacephalus*	Xeric	11	2.02	26.3	Benton Valley, CA, USA	Hinds and MacMillen (1985)
*Octodon degus*	Xeric	136.4	0.89	16.6	Fray Jorge National Park, Chile	Cortés et al. (2000)
*Perognathus fallax*	Xeric	19.6	3.03	17.3	Morongo Valley, CA, USA	Hinds and MacMillen (1985)
*Perognathus longimembris*	Xeric	8	2.08	25.8	Joshua Tree, CA, USA	Hinds and MacMillen (1985)

Abbreviations: EWL, evaporative water loss; *T*_a_, ambient temperature; MWP, metabolic water production; *T*_a_ @ MWP = EWL, ambient temperature at which metabolic water production equals evaporative water loss. Biogeographic regions refer to the collection localities or geographic regions of the populations from which the physiological data were obtained.

### Data analysis

Statistical analyses were performed using SPSS 26.0 (IBM, Armonk, NY, USA). Data were tested for normality and homogeneity of variance using the Kolmogorov–Smirnov and Levene tests, respectively. As no significant sex-dependent differences were found in the physiological parameters, data from both sexes were pooled. Changes in body mass, *T*_b_, food intake, and metabolic rate during the experimental period were analyzed using repeated-measures analysis of variance. Independent samples t-tests were used to compare body mass, *T*_b_, and mass-specific metabolic rates between groups at specific time points. Intergroup differences in overall metabolic rate and food intake were analyzed using analysis of covariance (ANCOVA). Organ masses and digestive tract measurements were analyzed using multivariate analysis of covariance (MANCOVA). When the overall MANCOVA was significant, further univariate analyses of covariance were performed. Body mass was used as the covariate in all analyses. Relationships between physiological variables were assessed using Pearson correlation analysis; prior to this, organ indices were corrected using linear regression residuals with day 7 body mass as the covariate. Regression analyses were employed to model the relationships between *T*_a_ and EWL, *T*_a_ and the MWP/EWL ratio, and body mass and EWL. For interspecific comparisons, log_10_-transformed EWL was analyzed using ANCOVA, with habitat type as a fixed factor and log_10_-transformed body mass as a covariate. Results are presented as mean ± SE, and statistical significance was set at *P* < 0.05.

## Results

### Effects of water deprivation on body mass and food intake

Prior to the experiment, no significant difference in body mass was observed between the control group (28.3 ± 1.1 g) and the water-stressed group (*t* = 0.32, *df* = 10, *P* = 0.75). Significant differences in body mass emerged starting from day 3, with the water-stressed group being 10.71% lower than the control group (*t* = 2.64, *df* = 10, *P* < 0.05). By day 7, the difference remained statistically significant (*t* = 3.14, *df* = 10, *P* = 0.01), with the water-stressed group weighing 13.52% less than the control. While the body mass of the control group remained stable throughout the experiment (*F*_(2,10)_ = 0.02, *P* = 0.98), the water-stressed group exhibited a sharp decline (*F*_(2,10)_ = 15.99, *P* < 0.01), showing a 12.90% decrease by day 7 compared with day 0 (Fig. 1A).

**Fig. 1:**
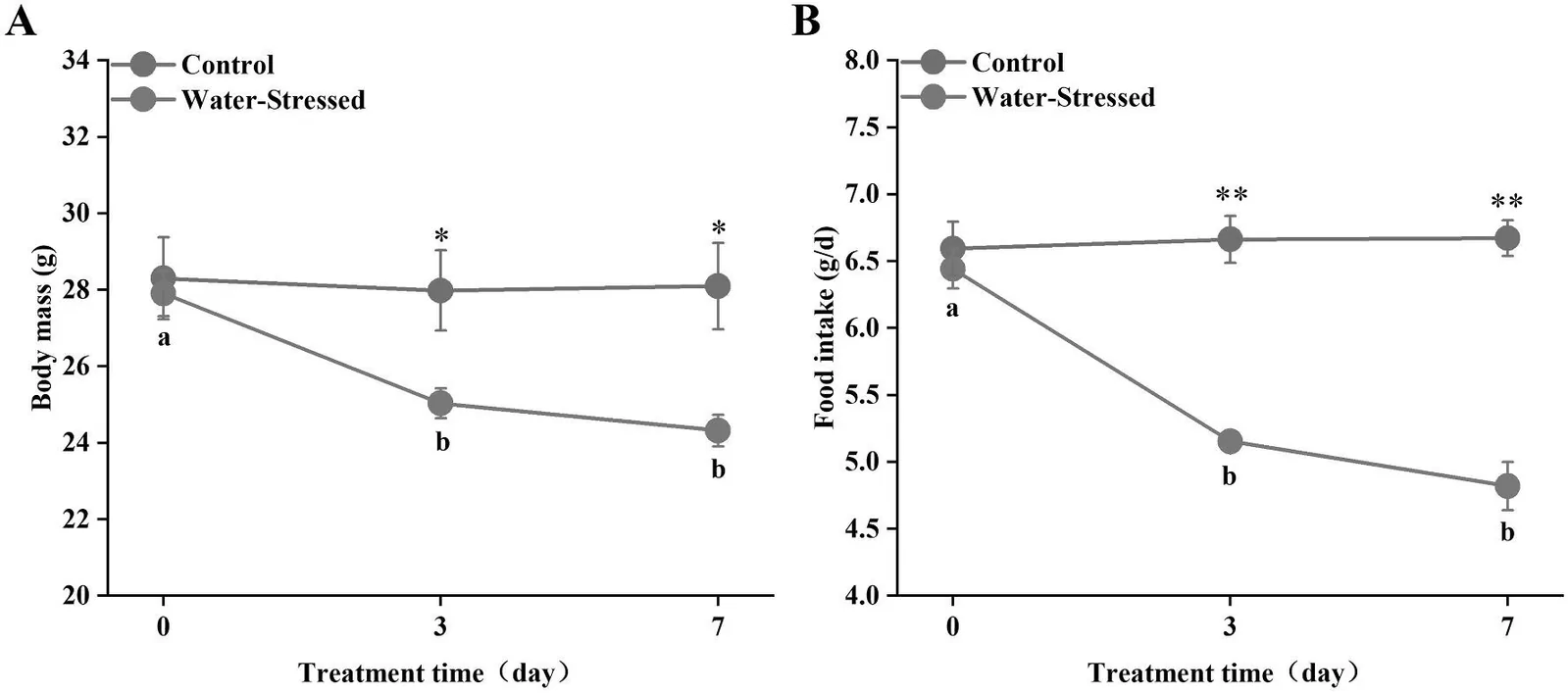
Effects of water deprivation stress on body mass (A) and food intake (B) in *A. chevrieri*. Different letters (a, b) denote significant differences (*P* < 0.05) among time points within the same treatment group, while identical letters represent no significant difference. Significant differences between the water-stressed and control groups are indicated by *P < 0.05 and **P < 0.01.

Regarding food intake, no baseline difference existed between the groups (*F*_(1,9)_ = 0.31, *P* = 0.59). From day 3 onward, food intake was significantly lower in the water-stressed group than in the control (Day 3: *F*_(1,9)_ = 33.61, *P* < 0.01; Day 7: *F*_(1,9)_ = 28.39, *P* < 0.01). On day 7, food intake in the water-stressed group was 4.82 ± 0.44 g, which was 27.74% lower than the control (6.67 ± 0.43 g). Within-group analysis showed a significant reduction in food intake for the water-stressed group (*F*_(2,10)_ = 38.94, *P* < 0.01), with a total decrease of 1.62 g by day 7 (Fig. 1B).

### Effects of water deprivation on metabolic rate

Initially, there were no significant differences in overall metabolic rate or BMR between the two groups (Overall: *F*_(1,9)_ = 0.03, *P* = 0.88; BMR: t < 0.01, *df* = 10, *P* = 1.00). By day 7, the BMR of the water-stressed group was significantly lower (by 11.47%) than that of the control group (*t* = 3.67, *df* = 10, *P* = 0.004). Over the course of the experiment, metabolic parameters in the control group remained stable. In contrast, the water-stressed group exhibited significant declines in both overall metabolic rate (*F*_(2,10)_ = 26.78, *P* < 0.01) and BMR (*F*_(2,10)_ = 4.21, *P* < 0.05), with day 7 values being 21.49% and 10.24% lower than day 0 baseline values, respectively (Fig. 2A, B).

**Fig. 2:**
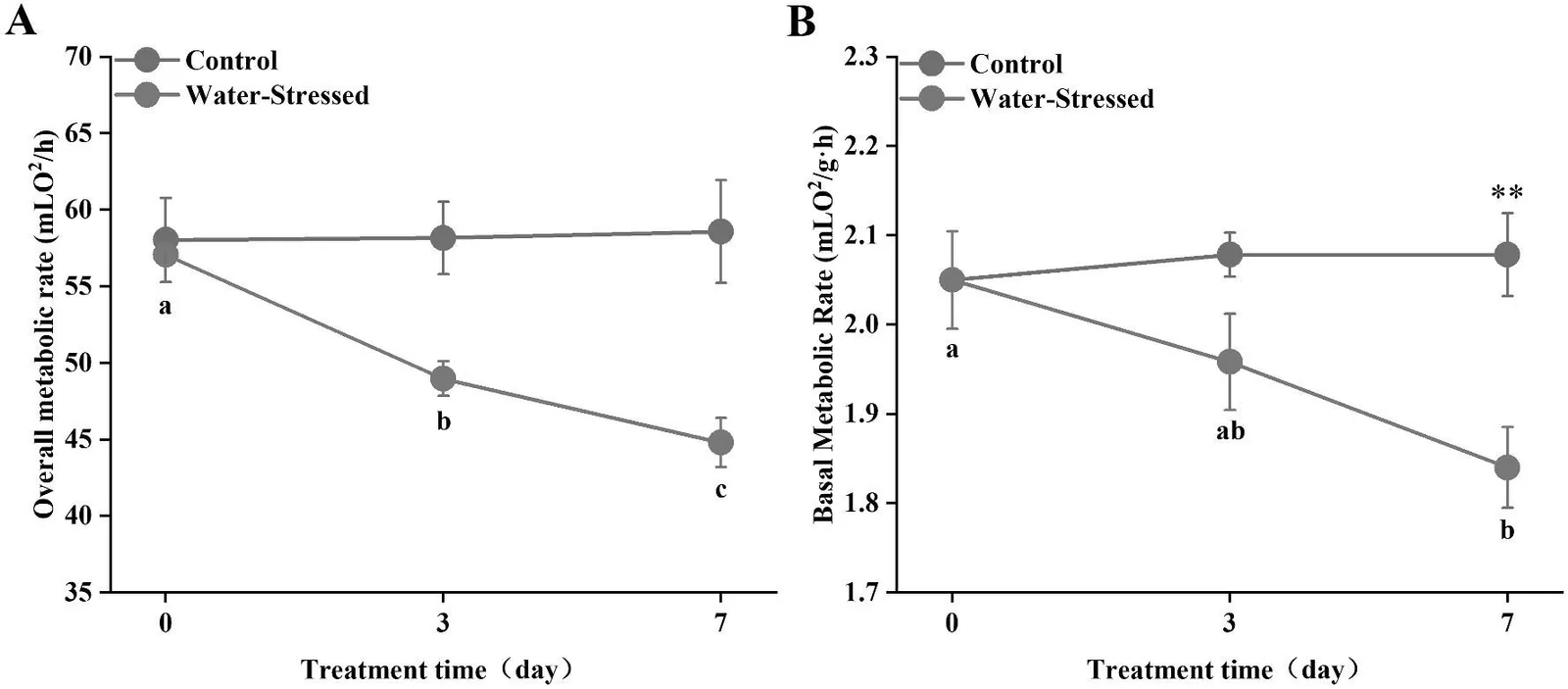
Effects of water deprivation stress on overall metabolic rate (A) and BMR (B) in *A. chevrieri*. Different letters (a, b, c) denote significant differences (*P* < 0.05) among time points within the same treatment group, while identical letters represent no significant difference. Significant differences between the water-stressed and control groups are indicated by *P < 0.05 and **P < 0.01.

### Effects of water deprivation on body and surface temperatures

No significant difference in *T*_b_ was found between the control and water-stressed groups prior to treatment (*P* = 0.10). Throughout the experimental period, the control group maintained a stable *T*_b_ (*F*_(2,10)_ = 0.04, *P* = 0.96), while the water-stressed group showed a significant decrease (*F*_(2,10)_ = 8.05, *P* < 0.01), dropping by 0.85°C from day 0 to day 7 (Fig. 3A). Surface temperature also decreased under water deprivation; infrared thermography revealed that heat dissipation in *A. chevrieri* was primarily concentrated in the cranial region, while tail temperatures remained notably low (Fig. 3B).

**Fig. 3:**
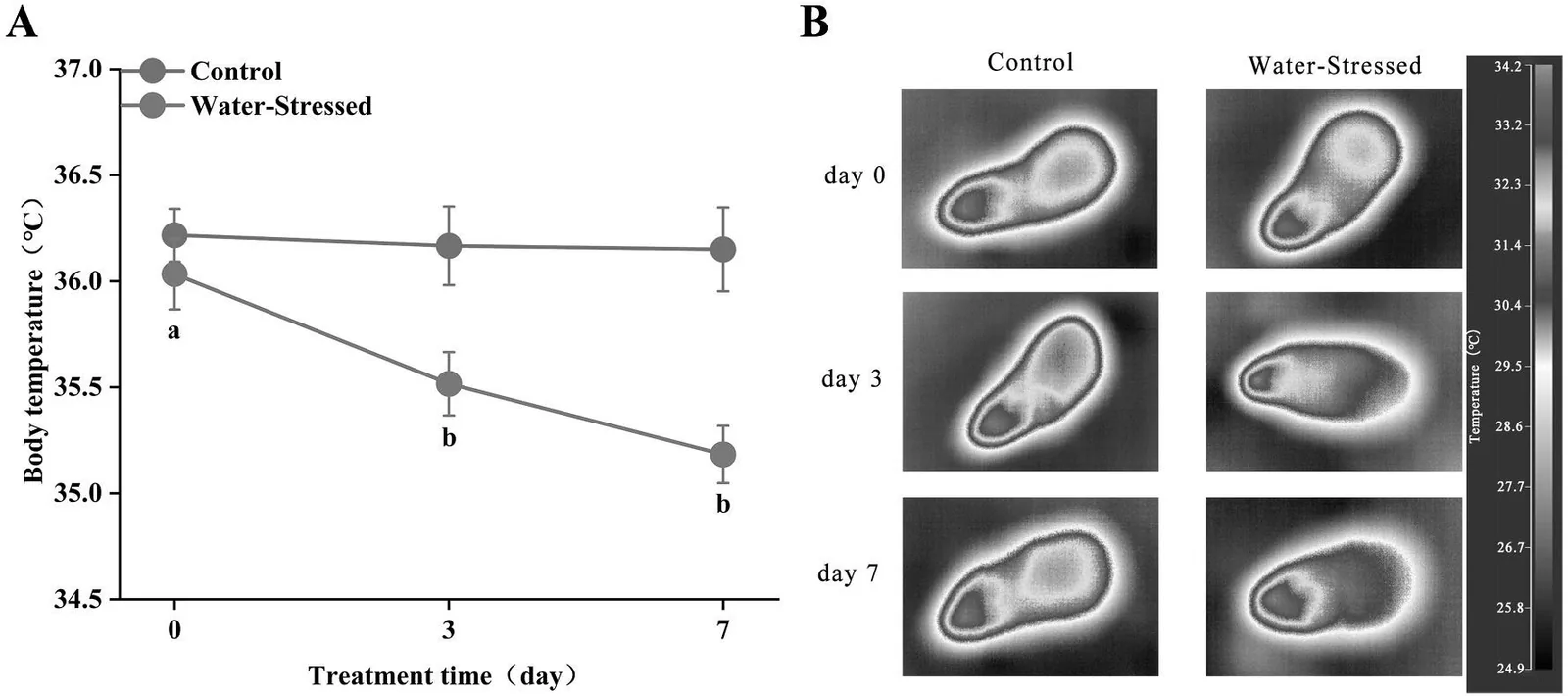
Effects of water deprivation stress on Tb (A) and surface temperature (B) in *A. chevrieri*. Different letters (a, b) denote significant differences (P < 0.05) among time points within the same treatment group, while identical letters represent no significant difference. Significant differences between the water-stressed and control groups are indicated by *P < 0.05 and **P < 0.01.

### Effects of water deprivation on digestive tract morphology

Water deprivation had a significant overall effect on digestive tract mass (Wilks’ λ = 0.15, *F*_(4,6)_ = 8.46, *P* < 0.05). After 7 days of water deprivation, significant differences in the mass of specific digestive tract segments were observed between the two groups. Compared with the control group, the small intestine mass in the water-stressed group was significantly reduced (*F*_(1,9)_ = 8.17, *P* < 0.05), whereas the large intestine mass was significantly increased (*F*_(1,9)_ = 8.88, *P* < 0.05). Specifically, small intestine mass decreased by 8.40%, while large intestine mass increased by 22.31% relative to the control group. In contrast, no significant differences were found in the mass of the stomach (*F*_(1,9)_ < 0.01, *P* = 0.99) or cecum (*F*_(1,9)_ = 0.02, *P* = 0.90) (Fig. 4A).

**Fig. 4:**
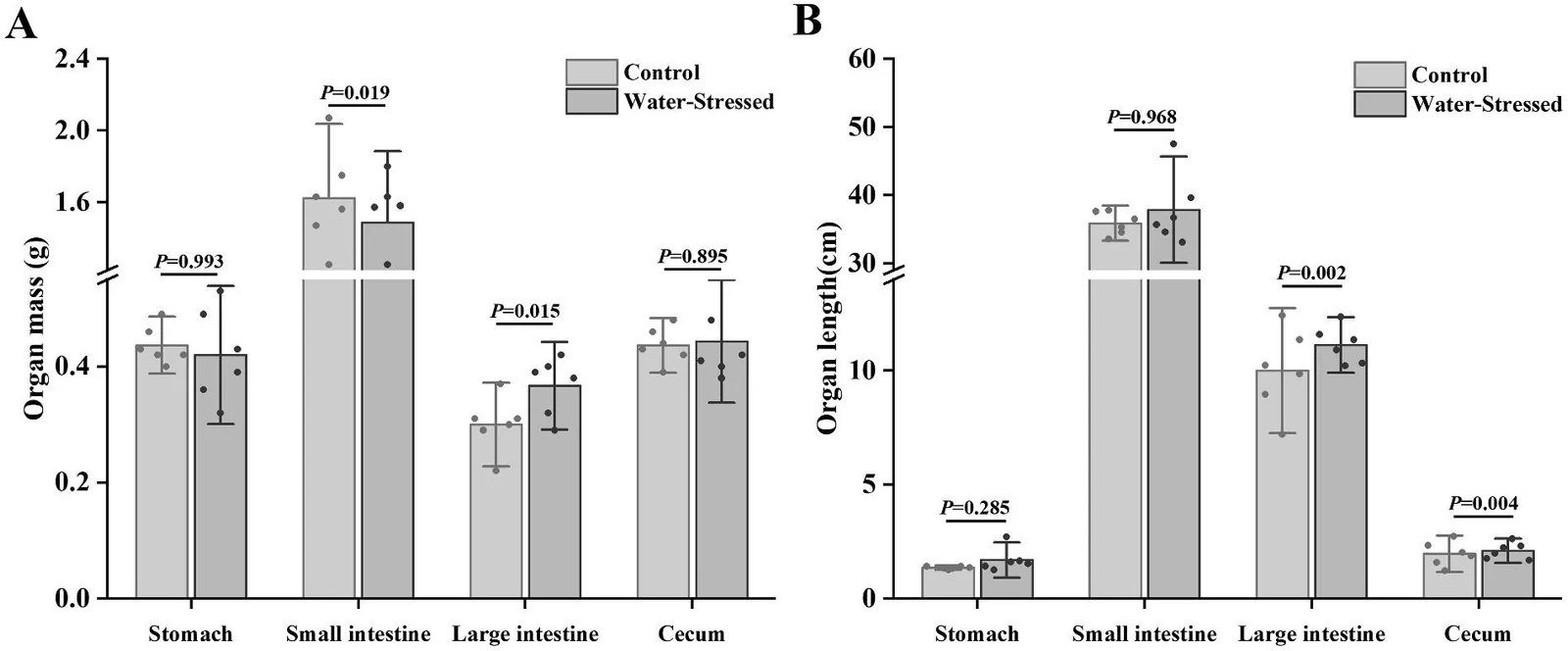
Effects of water deprivation stress on digestive tract mass (A) and length (B) in *A. chevrieri*. Scattered points represent individual measured values. *P* < 0.05 indicates a statistically significant difference between the water-stressed and control groups.

Digestive tract length also showed a significant overall effect (Wilks’ λ = 0.10, *F*_(4,6)_ = 13.10, *P* < 0.01). Following 7 days of water deprivation, no significant differences were detected between the control and water-stressed groups regarding stomach length (*F*_(1,9)_ = 1.29, *P* = 0.29) or small intestine length (*F*_(1,9)_ = 0.02, *P* = 0.97). However, significant differences were observed in the lengths of the large intestine and cecum. The large intestine length was significantly greater in the water-stressed group than in the control group (*F*_(1,9)_ = 17.89, *P* < 0.01), representing an 11.21% increase. Similarly, the cecum length was significantly increased in the water-stressed group (*F*_(1,9)_ = 14.89, *P* < 0.01), showing a 6.98% increase compared to the control (Fig. 4B).

### Effects of water deprivation on organ mass

The overall effect of water deprivation on organ mass did not reach statistical significance (Wilks’ λ = 0.19, *F*_(5,5)_ = 4.37, *P* = 0.07). Accordingly, univariate tests were not interpreted further. Across the measured organs, including the brain, heart, liver, lungs, and kidneys, absolute masses showed no clear overall shift between the control and water-stressed groups (Fig. 5).

**Fig. 5:**
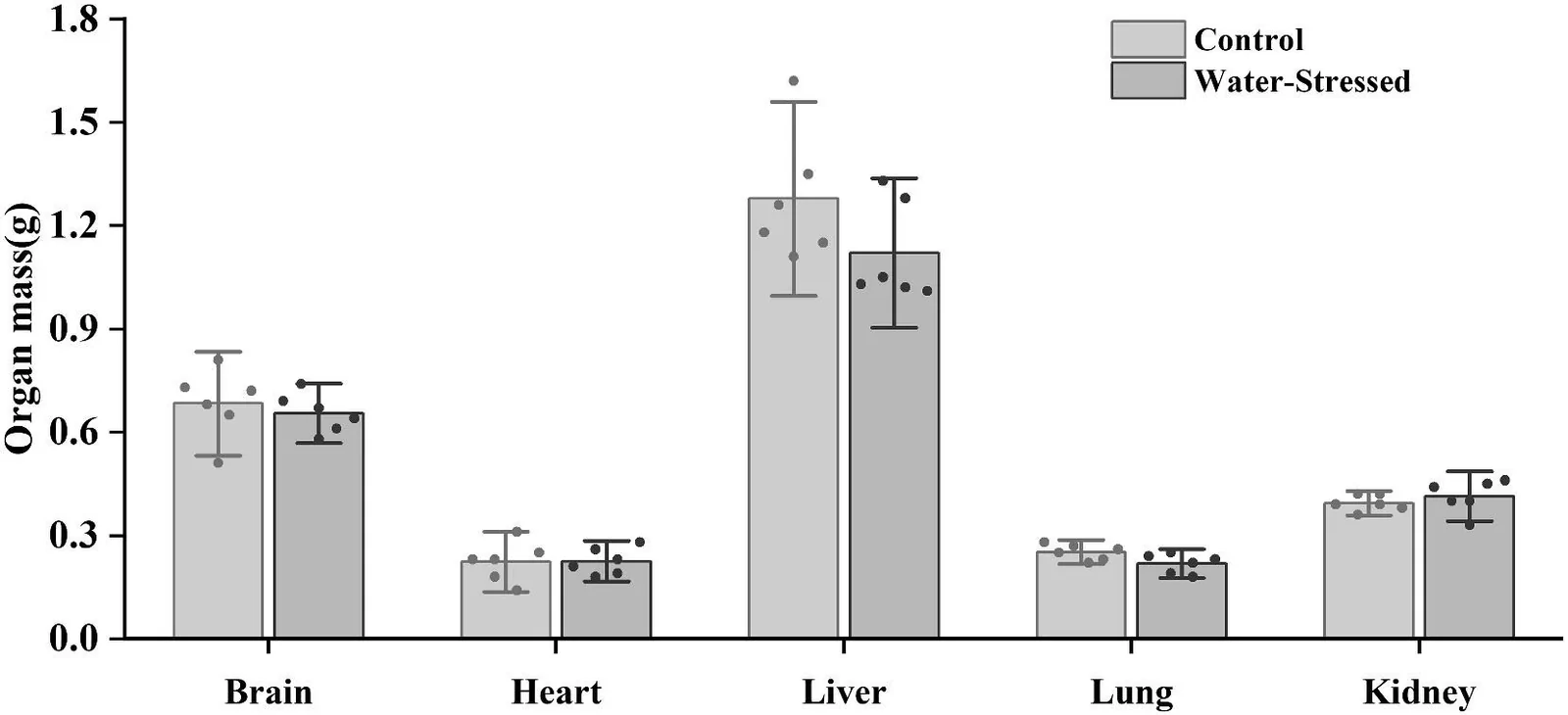
Organ masses of *A. chevrieri* under control and water-stressed conditions. Scattered points represent individual measured values. No significant overall differences were detected across organ masses between groups according to MANCOVA.

### Correlation analysis of physiological indicators

After water stress, body mass of *A. chevrieri* was significantly positively correlated with liver mass and small intestine mass (*P* < 0.05). Food intake was significantly positively correlated with BMR, stomach mass, and stomach length (*P* < 0.05), but significantly negatively correlated with lung, kidney, and large intestine mass (*P* < 0.05). BMR was significantly negatively correlated with kidney mass (*P* < 0.05) (Figs. 6 and 7).

**Fig. 6:**
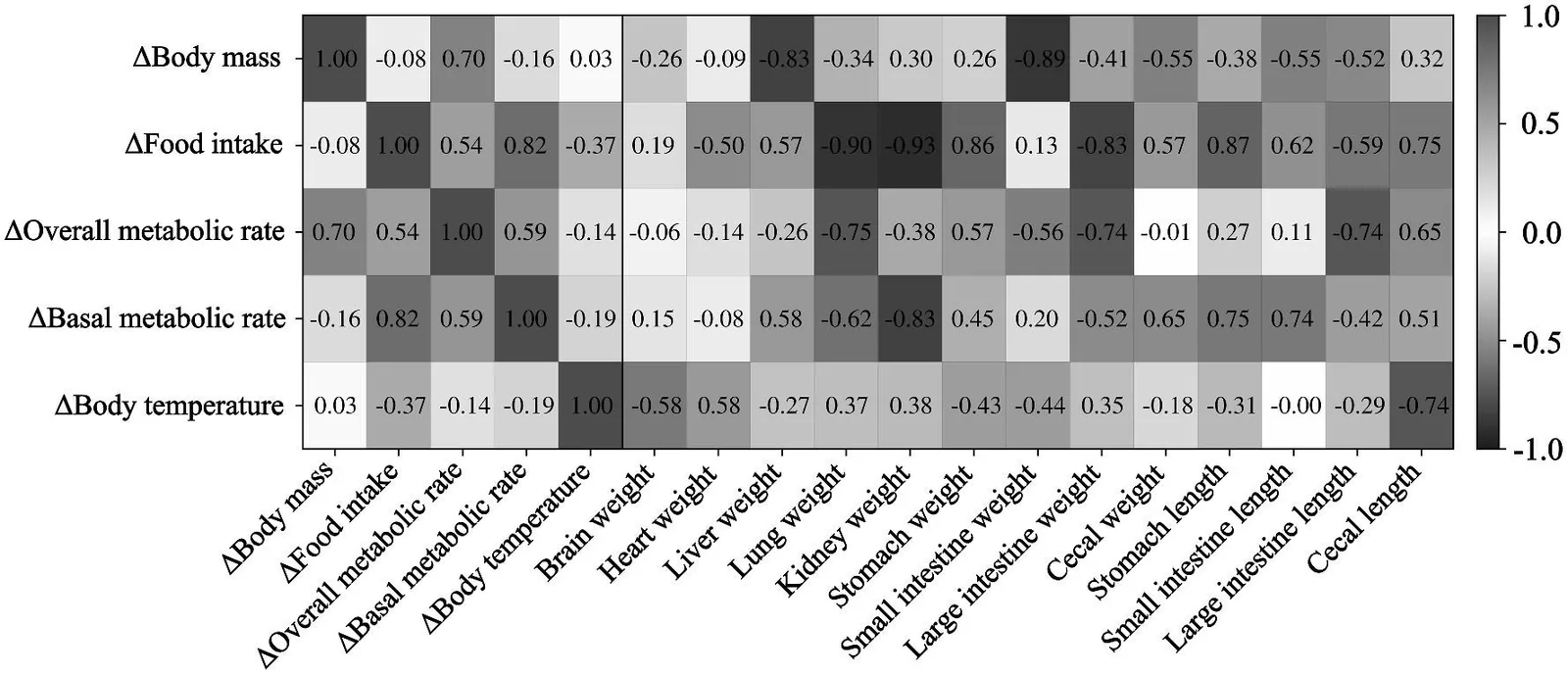
Heatmap of correlations between physiological indicators in *A. chevrieri* following water deprivation stress. The numbers within the squares represent the corresponding *r*-values, positive numbers represent positive correlation, negative numbers represent negative correlation. Δ represents the change in each indicator (Day 7 vs. Day 0).

**Fig. 7:**
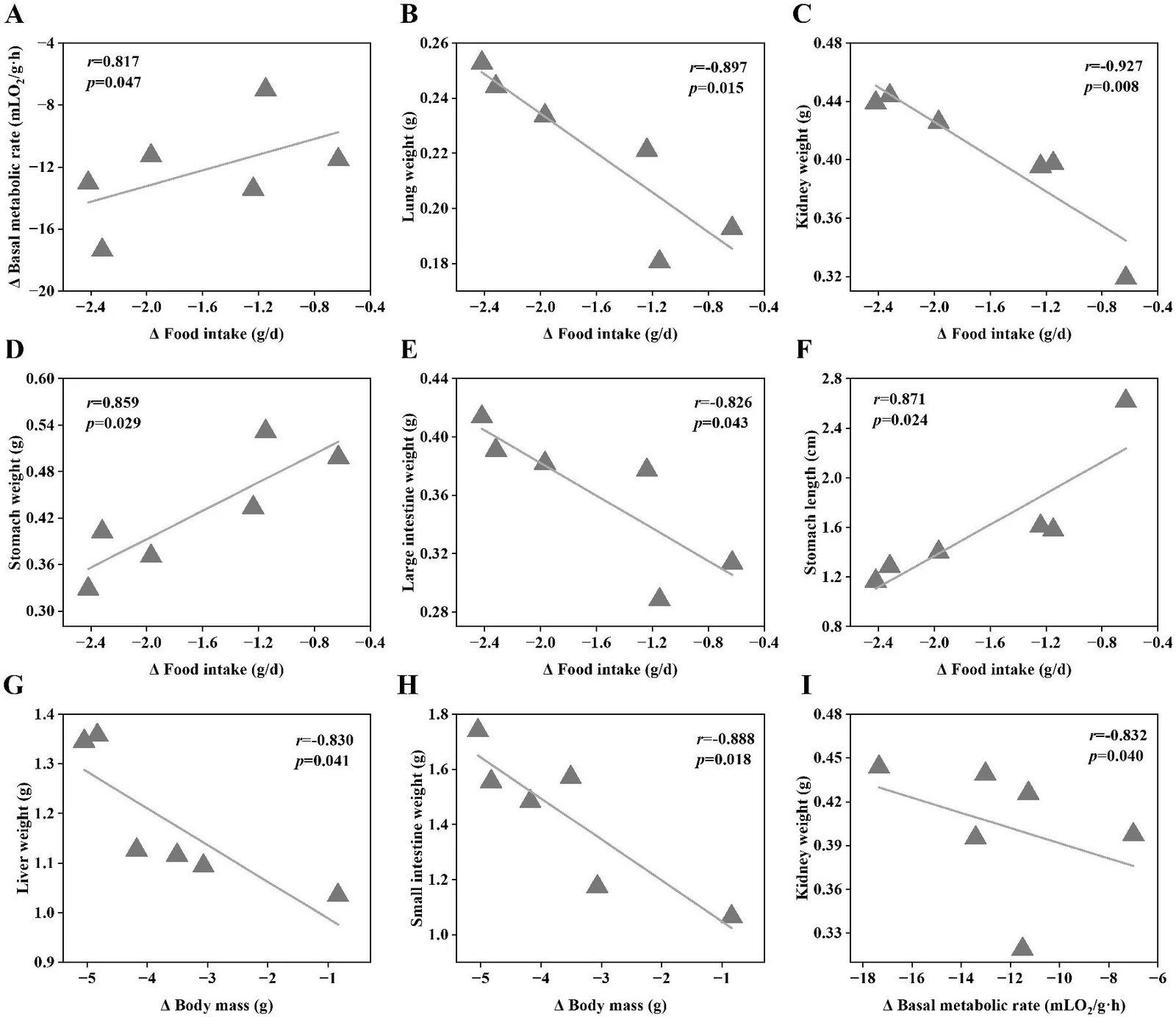
Linear correlation scatter plots between food intake, BMR, body mass, and organ indices after water deprivation stress. *r* denotes the Pearson correlation coefficient; *P* < 0.05 indicates a significant correlation, and Δ represents the change in each indicator (Day 7 vs. Day 0).

### Effects of water deprivation on EWL and MWP/EWL ratio

Within the *T*_a_ range of −5 to 20°C, EWL increased with rising *T*_a_ and showed significant positive correlations with *T*_a_ in both groups: control group, EWL = 1.70 + 0.10T_a_ (*r* = 0.98, *P* < 0.01); water-stressed group, EWL = 1.52 + 0.09T_a_ (*r* = 0.97, *P* < 0.01). Concurrently, the MWP/EWL ratio decreased with increasing *T*_a_ and showed significant negative correlations with *T*_a_: control group, MWP/EWL = 3.23 − 0.16T_a_ (*r* = −0.92, *P* < 0.01); water-stressed group, MWP/EWL = 5.24 − 0.29T_a_ (*r* = −0.85, *P* < 0.05). Within the *T*_a_ range of 20–35°C, EWL continued to increase overall, while MWP/EWL stabilized (*P* > 0.05). The *T*_a_ at which MWP/EWL = 1 was 16°C for the water-stressed group and 12.5°C for the control group (Fig. 8).

**Fig. 8:**
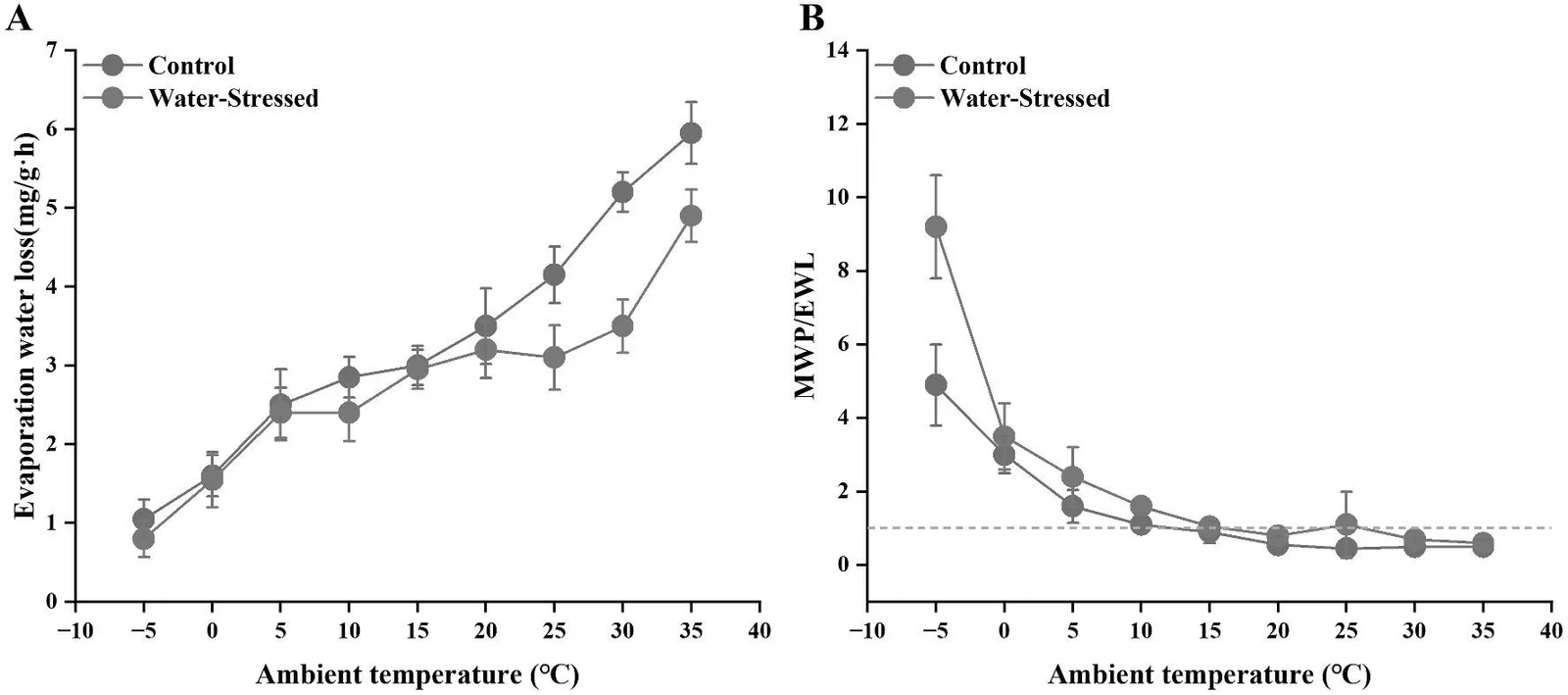
Relationship between Ta and EWL (A), and the ratio of MWP to EWL (B) in *A. chevrieri* following water deprivation stress. The dashed line in figure 8B represents the level where MWP/EWL = 1.

### EWL characteristics of *A. chevrieri* in comparison with mesic and xeric species

There was a significant difference in log₁₀EWL between xeric and mesic species after controlling for log₁₀ body mass (*F*_(1,22)_ = 34.06, *P* < 0.01), with xeric species exhibiting lower EWL. Comparative analysis of EWL among rodent species from different habitats revealed significant negative correlations between EWL and body mass in both habitat types: xeric species, log₁₀EWL = 0.71 − 0.36·log₁₀body mass (*r* = −0.88, *P* < 0.01); mesic species, log₁₀EWL = 1.02 − 0.40·log₁₀body mass (*r* = −0.72, *P* < 0.01) (Fig. 9A). The control-group *A. chevrieri* fell within the range for mesic species. The *T*_a_ @ MWP = EWL of water-stressed animals was higher than that of controls and approached values characteristic of certain xeric species (Fig. 9B).

**Fig. 9:**
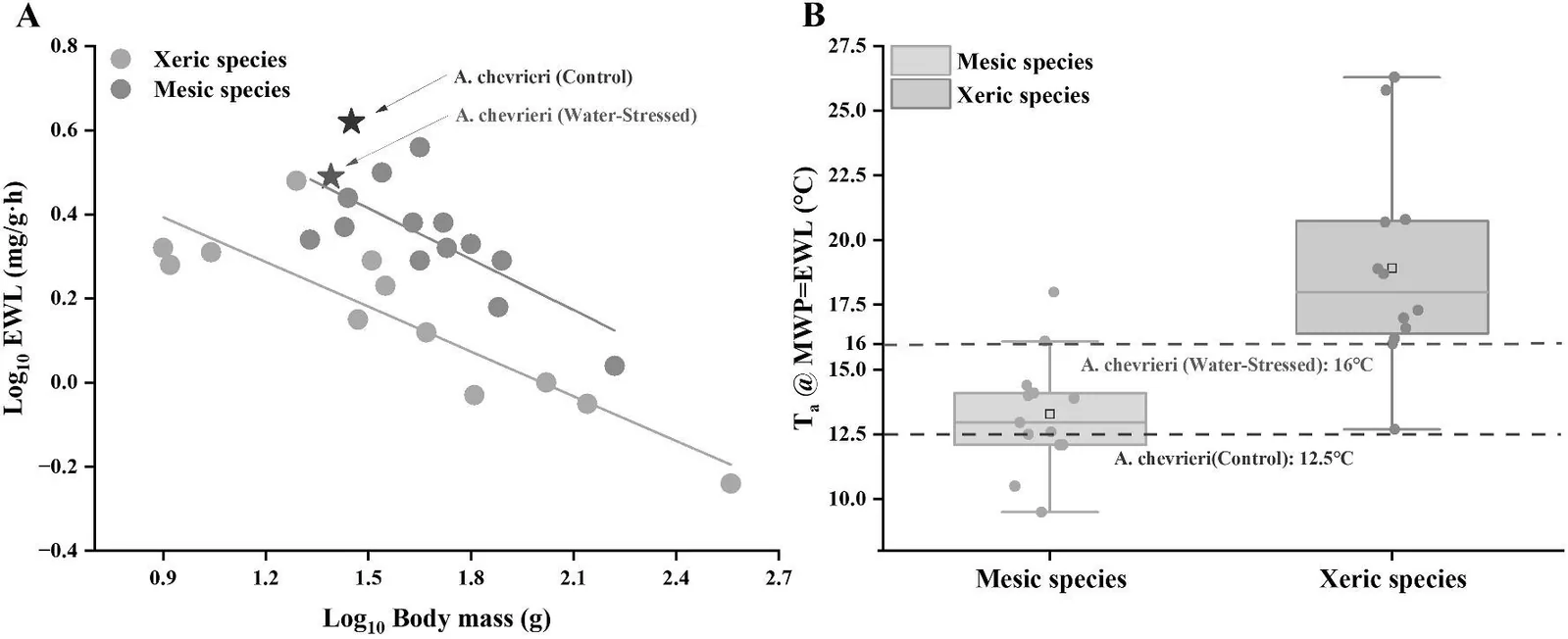
Relationship between body mass and EWL across different species (A), and *T*_a_ at the point of water balance across different species (B). Solid lines represent regression curves for species from different regional habitats. The dashed line indicates the *T*_a_ at the point of water balance for *A. chevrieri*.

## Discussion

In general, our findings supported the expectation that *A. chevrieri* relies on an integrated and synergistic physiological strategy to cope with acute water deprivation, shifting its overall phenotype toward minimizing both water and energy expenditure. As predicted, water-deprived individuals reduced food intake, body mass, BMR, and *T*_b_, while simultaneously remodeling hindgut morphology to enhance water reabsorption. Consistent with findings from other studies, this coordinated suppression of maintenance metabolism and heat dissipation appears to be a common adaptive response among small mammals facing resource limitation, although the specific mechanisms involved vary among species. A notable departure from generalized dehydration responses was the strict preservation of vital organ masses despite a marked 13.52% decline in overall body mass and substantial remodeling of the digestive tract. When evaluated within the mesic–xeric comparative context, these results indicate that although *A. chevrieri* exhibits baseline traits typical of a mesic species, its ability to shift *T*_a_ @ MWP = EWL from 12.5 to 16°C reflects an unexpectedly high degree of phenotypic plasticity, temporarily approximating the water-economy strategies typically observed in xeric-adapted rodents.

### Body mass and food intake

The regulation of body mass in small mammals is a fundamental strategy for survival and adaptation (Li and Wang 2005). As the basic medium for digestion, absorption, metabolism, and thermoregulation in animals, water availability directly affects feeding behavior and body mass maintenance (Golher et al. 2021). The breakdown and absorption of carbohydrates, fats, and proteins in food all depend on the participation of water. The excretion of metabolic waste also requires water as a carrier. Water shortage disrupts nutritional balance and metabolic homeostasis in animals, and then causes linked changes in food intake and body mass (Häussinger 1996; Fetissov and Meguid 2020). In this study, compared with the control group, body mass under water deprivation stress decreased by 10.71 and 13.52% on day 3 and day 7, respectively, while food intake decreased by 22.67 and 27.74% on day 3 and day 7, respectively. This is similar to the decrease in body mass of *Eothenomys miletus* under water deprivation stress (Yang and Zhu 2025), showing similar adaptation. However, in contrast to our findings, some species deviate from this pattern; for example, in *Notomys alexis*, food intake continued to increase and body mass also increased during water shortage (Takei et al. 2012). This may be related to the special water-saving strategy of this species in desert habitats. Other studies have shown that reduced feeding is directly related to the hydration state of the gastrointestinal tract, and dehydration of the digestive tract sends neural signals that suppress feeding (Schoorlemmer and Evered 2002). Therefore, *A. chevrieri* may reduce water demand in the digestive tract by decreasing food intake. It may also indirectly reduce metabolic intensity by decreasing nutrient intake, and then reduce heat dissipation-related water loss caused by metabolic heat production. The decrease in body mass may result from the combined effects of reduced food intake and metabolic expenditure. Reduced nutrient accumulation caused by insufficient food intake, together with body mass loss caused by the breakdown of stored substances for energy supply, may represent an adaptive strategy adopted by *A. chevrieri* to reduce survival costs under resource-limited conditions.

### Metabolic rate regulation

Metabolic rate is the dynamic basis of animal life activities and encompasses core indices such as overall metabolic rate and BMR. Water is indispensable in these processes, participating in everything from the oxidative breakdown of nutrients to the synthesis and utilization of ATP (Thornton 2010; Jéquier and Constant 2010; Popkin et al. 2010). The results of this study showed that overall metabolic rate and BMR of *A. chevrieri* in the water-stressed group decreased significantly over time. On day 7, BMR decreased by 10.24% relative to the initial value and was significantly lower than that of the control group. This aligns with our expectations and is highly consistent with findings from food restriction studies. When food intake of *A. chevrieri* is restricted, BMR decreases significantly (Liu et al. 2010). This suggests that whether through direct energy restriction or indirect reduction in intake due to water stress, *A. chevrieri* downregulates maintenance thermogenesis to reallocate its energy budget. By lowering metabolic rates and heat production, the species may reduce surface temperatures and the water required for evaporative cooling (Walsberg 2000). Interestingly, this strategy contrasts with findings in *Eutamias merriami*, where *T*_b_ and oxygen consumption rate remain nearly unchanged under long-term water restriction, as that species relies more on renal and behavioral regulation (Wunder 1970). In contrast, *A. chevrieri* appears to rely more on lowering overall metabolic level to cope with water deprivation stress. This may be related to its high-altitude environment, where it normally maintains a relatively high metabolic rate to deal with low temperature (Zhu et al. 2012; Gao and Zhu 2024). When water suddenly becomes limited, continued maintenance of a high metabolic rate would clearly increase water loss, so metabolic suppression may be more advantageous. Correlation analysis further showed that food intake was significantly positively correlated with BMR in *A. chevrieri* after water deprivation stress. This suggests that the decrease in metabolic rate under water deprivation stress may be both an active water-saving strategy and, in part, a result of reduced substrate supply and lower digestive costs caused by reduced food intake.

### Regulation of *T*_b_ and surface heat dissipation

Animals maintain a dynamic energy balance by adjusting their physiological and ecological traits in response to changing environments (Stawski and Simmonds 2021). *T*_b_ is a critical physiological indicator and is essential for energy regulation in animal energetics (Ayres 2020). Maintaining homeothermy in mammals requires continuous energy expenditure, while evaporative cooling serves as an unavoidable pathway for water loss (Schmidt-Nielsen 1997; Robertshaw 2006). Previous research has shown that *A. chevrieri* reduces *T*_b_​ to lower energy consumption during energetic challenges (Wang et al. 2007). In this study, *T*_b_ of *A. chevrieri* in the water-stressed group decreased by 0.85°C relative to the initial value on day 7, indicating that water deprivation reduced *T*_b_. The results of this study are supported by reports of hypothermia in *Peromyscus eremicus* and *Sminthopsis macroura* (Song and Geiser 1997; Blumstein and MacManes 2023), which may represent an adaptation to water-deprived environments. By reducing metabolic rate and limiting EWL, *T*_b_ decreases and the energy expenditure required to maintain a constant *T*_b_ is reduced, thereby optimizing energy allocation. We also observed a decrease in surface temperature, with heat dissipation concentrated in the cranial region, whereas tail surface temperatures remained relatively low. The tail functions as a critical “thermal window” in rodents, playing a core role in their adaptation to multiple environmental stressors. Recent research has shown that under extreme conditions, congeneric wild mice (*Apodemus* spp.) not only undergo severe peripheral vasoconstriction to lower tail temperature but also adopt a “tail-belting” behavior to restrict energy loss (Stryjek et al. 2024). In light of the surface temperature distribution observed in our study, it is likely that water restriction modifies peripheral vasoconstriction patterns, thereby reducing the total evaporative surface area while preserving the relative temperature stability of core functional areas (Kregel et al. 1988). These results suggested that *A. chevrieri* can cope with water-deprived conditions by actively reducing core temperature and lowering the demands for heat production and heat dissipation. This drop also reduces respiration rates, further minimizing water loss during gas exchange. This is a selective regional heat dissipation strategy to enhance water-use efficiency in response to climate change.

### Digestive tract remodeling

Phenotypic plasticity of the digestive system is an important mechanism for small mammals to cope with environmental stress (Wang et al. 1995). In rodents, the morphology of different digestive tract segments is closely related to their specific physiological functions and resource requirements. Meeting our expectations for structural adaptations that enhance water reabsorption, the gut morphology of *A. chevrieri* showed significant changes after water deprivation stress. Small intestine mass decreased by 8.40%, large intestine mass increased by 22.31%, large intestine length increased by 11.21%, and cecum length increased by 6.98%. The decrease in small intestine mass may be related to an active downregulation of digestive and absorptive functions, which would reduce energy and water use during digestion and match the observed decreases in food intake and metabolic rate. In contrast, the enlargement of the large intestine and cecum reflects a strategy to enhance microbial fermentation and water reabsorption. As the main site for water recovery, the morphological expansion of the large intestine can increase fecal water-holding time and reabsorption area. Cecal fermentation may produce volatile fatty acids and metabolic water through microbial metabolism. With reduced food intake, more energy is obtained by enhancing cecal fermentation function. Correlation analysis showed that after water deprivation, body mass was significantly positively correlated with small intestine mass, and food intake was significantly negatively correlated with large intestine weight. Combined with decreased food intake and metabolic rate, it can be inferred that under water deprivation conditions, *A. chevrieri* reduces maintenance costs by reducing metabolically expensive small intestine tissue. At the same time, moderately extending and increasing the large intestine and cecum improves chyme retention time in the hindgut and water reabsorption capacity, thereby maximizing water and nutrient recovery at lower feeding levels and producing drier feces.

### Changes in visceral organ mass

Water is essential for cellular metabolism. Lack of water usually causes coordinated physiological and morphological changes at the organ level to maintain internal homeostasis (Schmidt-Nielsen 1997; Donald and Pannabecker 2015). Studies have shown that under limited resources, animals tend to give priority to vital organs and reduce resource allocation to non-essential tissues, thereby ensuring overall survival (McCue 2010). In the present study, dehydration had no significant overall effect on the mass of the core visceral organs, including the brain, heart, liver, lungs, and kidneys. This strict resource prioritization deviated from the severe mass reductions observed in the rest of the body; despite a significant reduction in overall body mass and marked remodeling of the digestive tract, the absolute masses of these vital organs remained completely stable. This indicated that under short-term water deprivation stress, *A. chevrieri* adopted a stringent resource prioritization strategy. Rather than sacrificing vital organ tissues, the animal relied on whole-body metabolic suppression, hypothermia, and hindgut plasticity to reduce water and energy expenditure. During acute dehydration, maintaining the structural integrity of the brain, heart, and liver may provide a structural basis for sustaining essential physiological functions.

### EWL and MWP/EWL

In small mammals, EWL is an important route of body water loss. This process is influenced by *T*_a_, humidity gradients, ventilation volume, and heat dissipation strategies (Robertshaw 2006; Baldo et al. 2016). EWL typically increases as *T*_a_ rises or when the need for heat dissipation grows (Baldo et al. 2015). Conversely, MWP is generated through the oxidation of nutrients. The MWP/EWL ratio is a key indicator of water regulation efficiency in rodents (Bozinovic and Gallardo 2006). In the current study, *T*_a_ at which MWP/EWL = 1 shifted from 12.5°C in the control group to 16°C in the water-stressed group, indicating that dehydration changes the temperature response of water balance and improves water maintenance under warmer conditions by suppressing EWL or altering substrate use (MacMillen and Hinds 1983; Cortés et al. 2000). Further cross-species comparison showed that, based on the analysis of EWL and *T*_a_ @ MWP = EWL in 26 rodent species from different habitats, xeric species had lower EWL than mesic species, and the EWL values of both the water-deprived and control groups of *A. chevrieri* fell within the range of species from humid habitats. Xeric species also had higher *T*_a_ @ MWP = EWL values than mesic species (Fig. 9A). The *T*_a_ @ MWP = EWL value of the control group of *A. chevrieri* fell within the range of species from humid habitats, while the dehydration group showed a higher value and shifted toward the range of typical xeric species (Fig. 9B). Synthesizing these findings with the broader literature, it becomes clear that although *A. chevrieri* is fundamentally a mesic species, its response strategy reflects remarkable adaptive plasticity. Under water deprivation stress, it effectively shifts its water-economy phenotype toward a more xeric-like strategy by relying on a flexible, multi-tiered physiological defense mechanism. This mechanism integrates metabolic depression, targeted hindgut enlargement, and regulated hypothermia, thereby helping the species maintain survival during the increasingly severe seasonal droughts of the Hengduan Mountains.

## Conclusion

This study demonstrated that *A. chevrieri* responds to short-term water deprivation through an integrated physiological strategy that couples reduced energy intake and expenditure, regulated hypothermia, and hindgut morphological remodeling to enhance water recovery. Cross-species comparisons indicated that, although this species generally exhibits a mesic water-economy phenotype, it possessed sufficient plasticity to shift toward xeric-like water conservation under acute stress. These findings deepen the understanding of survival and adaptive strategies of *A. chevrieri* and provide a physiological basis for predicting population-level responses to increasing seasonal aridity in the Hengduan Mountains. Nevertheless, the study was limited by its short duration, modest sample size, and laboratory setting. Future research should employ long-term field monitoring, reproductive fitness assays, and mechanistic studies to determine whether this plasticity can sustain populations under the escalating drought regimes predicted for southwestern China.

## Data Availability

Data and raw photographs are available from the corresponding author upon reasonable request.
